# Finite Element Method and Von Mises Investigation on Bone Response to Dynamic Stress with a Novel Conical Dental Implant Connection

**DOI:** 10.1155/2020/2976067

**Published:** 2020-10-07

**Authors:** Luca Fiorillo, Marco Cicciù, Cesare D'Amico, Rodolfo Mauceri, Giacomo Oteri, Gabriele Cervino

**Affiliations:** ^1^Department of Biomedical and Dental Sciences, Morphological and Functional Images, University of Messina, Policlinico G. Martino, Via Consolare Valeria, 98100 Messina, Italy; ^2^Department of Surgical Oncological and Oral Sciences, University of Palermo, 90127 Palermo, Italy

## Abstract

The bioengineering and medical and biomedical fields are ever closer, and they manage to obtain surprising results for the development of new devices. The field of simulations and studies in silica has undergone considerable development in recent years, favoring the advancement of medicine. In this manuscript, a study was carried out to evaluate the force distribution on the implant components (In-Kone® Universal) and on the peri-implant tissues subjected to loading. With the finite element analysis and the Von Mises method, it was possible to evaluate this distribution of forces both at 0 degrees (occlusal force) and at 30 degrees; the applied force was 800 N. The obtained results on this new type of connection and on all the implant components are satisfactory; the distribution of forces appears optimal even on the peri-implant tissues. Surely, studies like this help to obtain ever more performing devices, improving both the clinic and the predictability of rehabilitations.

## 1. Introduction

The finite element technique, also known with the acronym FEM (finite element method) has historically established itself for the study of structural phenomena related to stiffness, strength, and elastic stability of bodies. Thanks to the experience gained over the years, manufacturers are able to perform structural calculations on complete machines, boats, cars, aeronautical structures, consumer goods, and industrial plants. Modeling and computational analysis are giving medical engineering a significant competitive advantage by reducing risks, lowering costs, and accelerating innovation [1–5]. FEM analysis is a computer simulation technique applicable to many engineering sectors. The FEM analysis allows to describe a real system accurately and reliably, in order to obtain the physical quantities of interest [6–12]. Depending on the applications, these quantities could be displacements, temperatures, stresses, deformations, electric/magnetic fields, pressure, etc. The advantage of integrating FEM analysis into the design method lies in the possibility of studying complex physical phenomena that could otherwise be addressed with an experimental approach, more expensive. FEM allows us to identify any problems before the prototype is even made and therefore to review the design quickly and economically. In addition to identifying malfunctions, with FEM, it is possible to optimize a structure by removing excess material and improving weight distribution. Applied to fluid dynamics, the same methodology allows to limit pressure and flow losses by refining the profiles of grids, fans, and pipes. In most biomechanical finite element analyses, the linear elastic behavior of biological tissues is assumed [13, 14].

The analyst's true ability lies in building a model that simulates reality well without exceeding in the finesse of discretization in points of little structural interest and in identifying the constraints and loads that reflect the physics of the problem. The application of this method therefore requires a good basic theoretical knowledge that allows a targeted choice of the elements to be used, in relation to the analysis to be conducted, and a critical interpretation of the results obtained in light of the limitations and approximations of the method. It is also necessary to pay constant attention to experimental analyses that allow validating the hypothesized approximations. The bioengineering and biomedical investigations are of great help in the prosthetic field and especially in the dental field [7, 15]. Considerable studies have been conducted to evaluate the stress of dental implants on the different biomechanical forces present in the oral cavity.

Stages of the realization of a finite element model are as follows:

- Preparation of the geometric model
- Discretization of the entire volume in finite elements (tetrahedra or parallelepipeds)
- Assignment of the mechanical properties of the materials
- Identification of loads and constraint points
- Choice of the type of solution (static or dynamic analysis, linear or nonlinear, etc.)
- Analysis of the results [16, 17]

The aim of this study is to evaluate the biomechanical behavior of the In-Kone® dental implant connection. This device has been studied under the action of the mandibular force that is exercised during chewing cycles. The study was divided into three steps:

- The first step was the reverse engineering of the prosthesis, which allowed the transformation of a STL scan into a three-dimensional CAD model
- The second step was the creation of the mechanical model, with applications of the boundary conditions of loads and constraints
- Finally, results on mechanical behavior are obtained, i.e., on the distribution of stresses in the three prosthodontics [18–20]

The null hypothesis was assumed that there are no clinical differences between in silica studies and in vivo conditions.

## 2. Materials and Methods

The first step in setting up the FEM was to perform Reverse reverse Engineering engineering of the In-Kone® Universal prosthesis so as to obtain a CAD CAD-type file from the STL source file, and finally, a rendering was done on the CAD reconstructed through the Keyshot® software.

**Figure 1:**
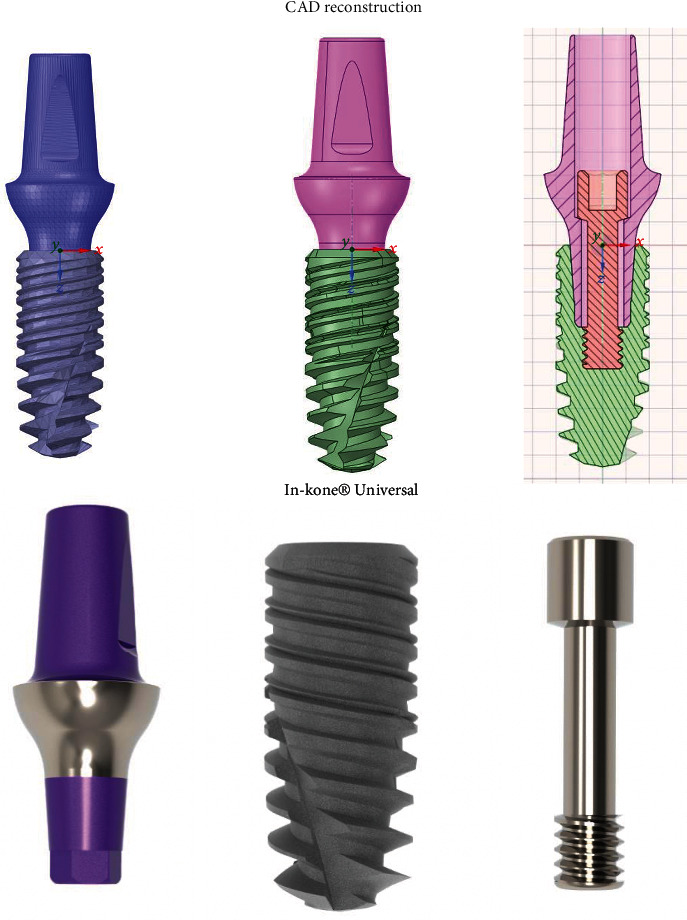
Reverse engineering of prostheses.

**Figure 2:**
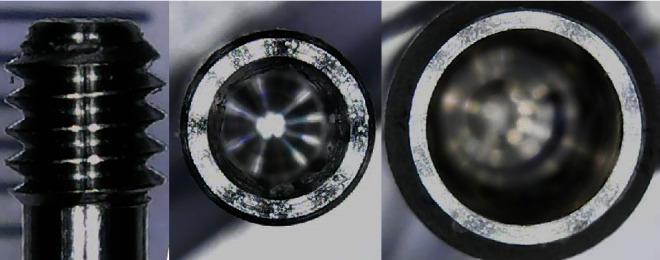
Acquisition of missing measures from the real component.

The missing measurements were acquired using a digital microscope on the prosthesis from the real. The supplied STLs have a low resolution as can be seen in Figure 1; moreover, the retention screw did not respect the real dimension measured through a gauge on the piece from the truth. This difficulty has been overcome by reconstructing the geometry of this component through the use of a digital microscope. In Figure 2, there is an example of measurements taken on the real pieces, which were missing in the STL files.

**Figure 3:**
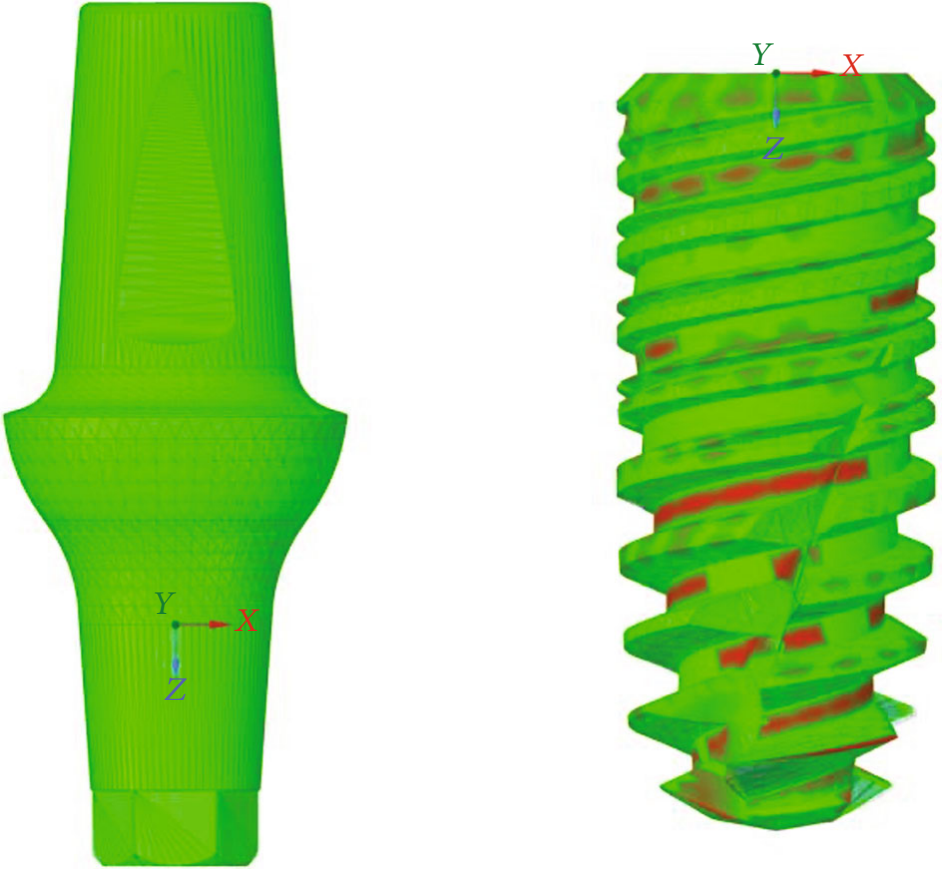
Maximum deviation equal to 0.09 mm of the CAD with respect to the STL.

The reverse was carried out maintaining maximum deviations with respect to the geometry of the STL file of the order of a tenth of a millimeter (Figure 3).

Figure 1 shows reverse engineering and a sagittal section, along the *y* axis, and rendering of the three components.

The FEM simulation was performed through the Siemens NX Nastran® software. The properties of the materials have been specified in terms of Young's modulus, Poisson's ratio, and density. In particular, the titanium alloy Ti6Al4V was considered homogeneous, linear, and isotropic, while the cortical and cancellous bone tissues were considered as orthotropic (Table 1). The data obtained in the literature were used as mechanical characteristics of the materials.

**Figure 4:**
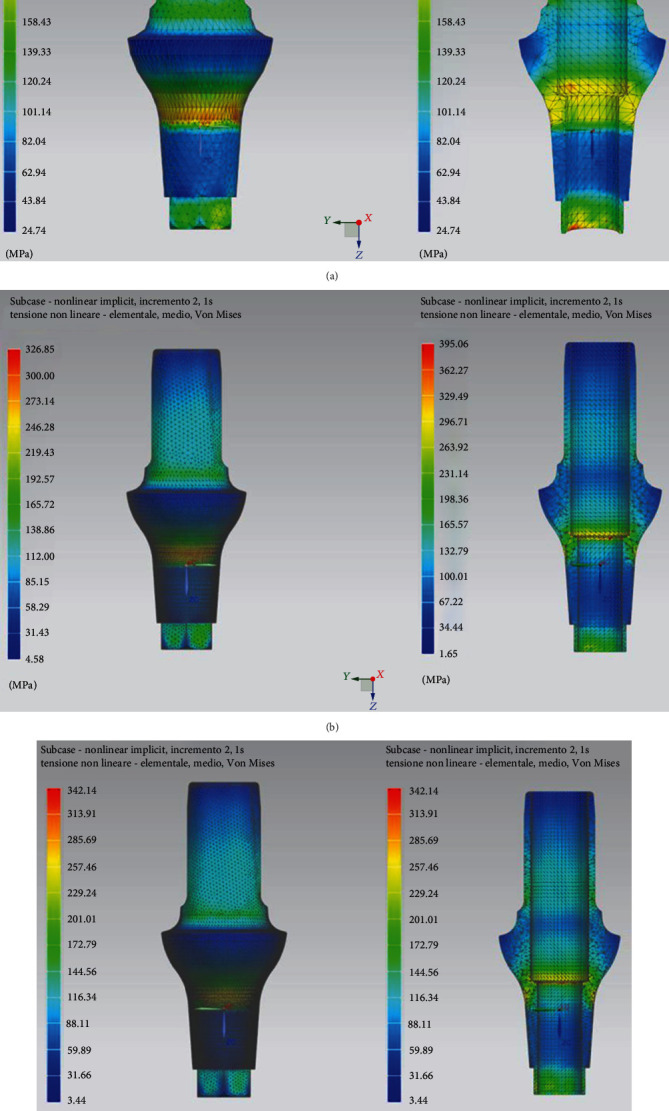
Mesh sensitivity study 0.4 mm elements (a), 0.2 mm (b), and 0.1 mm (c).

The mesh was made with 4-node solid tetrahedral elements, of the CTETRA 4 type; this allows considerable computational resource savings compared to the 10-node tetrahedral. The cell size fell on 0.2 mm elements. This value was chosen after performing the convergence analysis of the mesh (Figure 4).

This study makes it possible to find the right compromise between simulation calculation speed and reliable stress values. As the size of the elements changes, the stress converges to a value that remains stable even at 0.2 mm. In Table 2, it is possible to observe the estimated error taking as a reference to the element size of 0.1 mm.

The chosen value of 0.2 mm induces a 4.47% error on the stress value, which can be considered an acceptable compromise as less than 5%.

**Figure 5:**
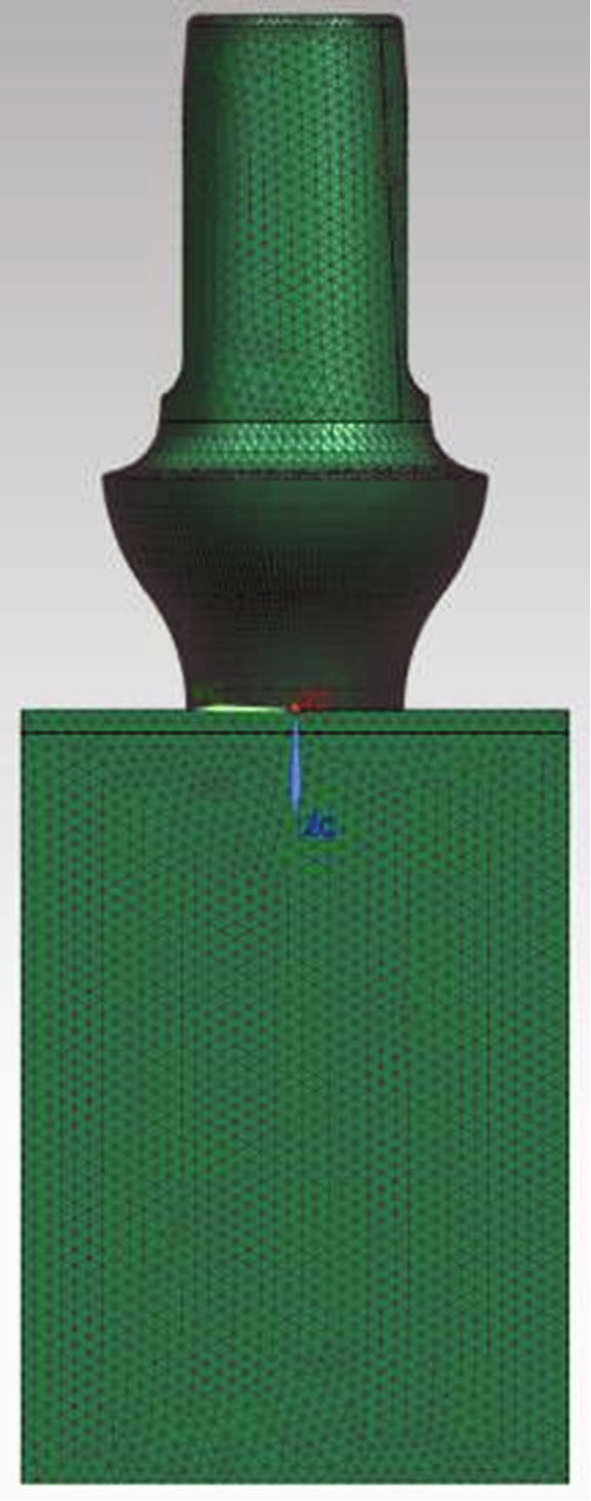
Prosthetic mesh.

Figure 5 shows the prosthesis mesh implanted on a parallelepiped (the latter mechanically characterized as cortical and cancellous bone tissue), with an element size of 0.2 mm.

### 2.1. Zero-Degree Boundary Conditions

**Figure 6:**
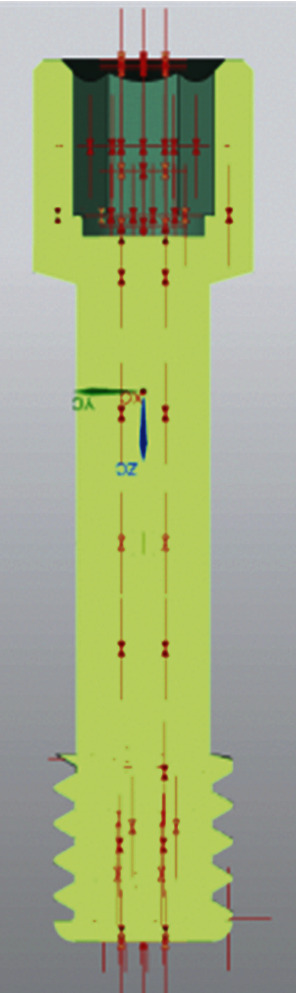
Preload on a screw.

The boundary conditions of the system concern the application of the preload force of the internal tightening screw and of the maximum chewing force on the prosthetic stump. The force value of the preload 430 N is relative to a tightening torque of 15 Ncm. This force was calculated through the following empirical formula:

(1) $$\begin{array}{c} M=KDP, \end{array}$$

where *M* is the tightening torque (expressed in Nmm), *K* is a global coefficient that takes into account the friction coefficients on the thread, diameter, and pitch of the screw (in the case under examination, it is equal to 0.2), *D* is the diameter of the thread (expressed in mm), and *P* is the axial preload applied to the screw (expressed in N). To model the clamping force, the practical NX Nastran® tool was used to apply this force to the 3D bolt or screw model. Its functioning is easy to understand; at the beginning of the simulation, the software first applies the preload force gradually up to the maximum value, then applies the remaining load acting on the model [15, 21, 22].  Figure 6 shows the section of the internal screw with the traction forces applied by the preload on the geometry.

**Figure 7:**
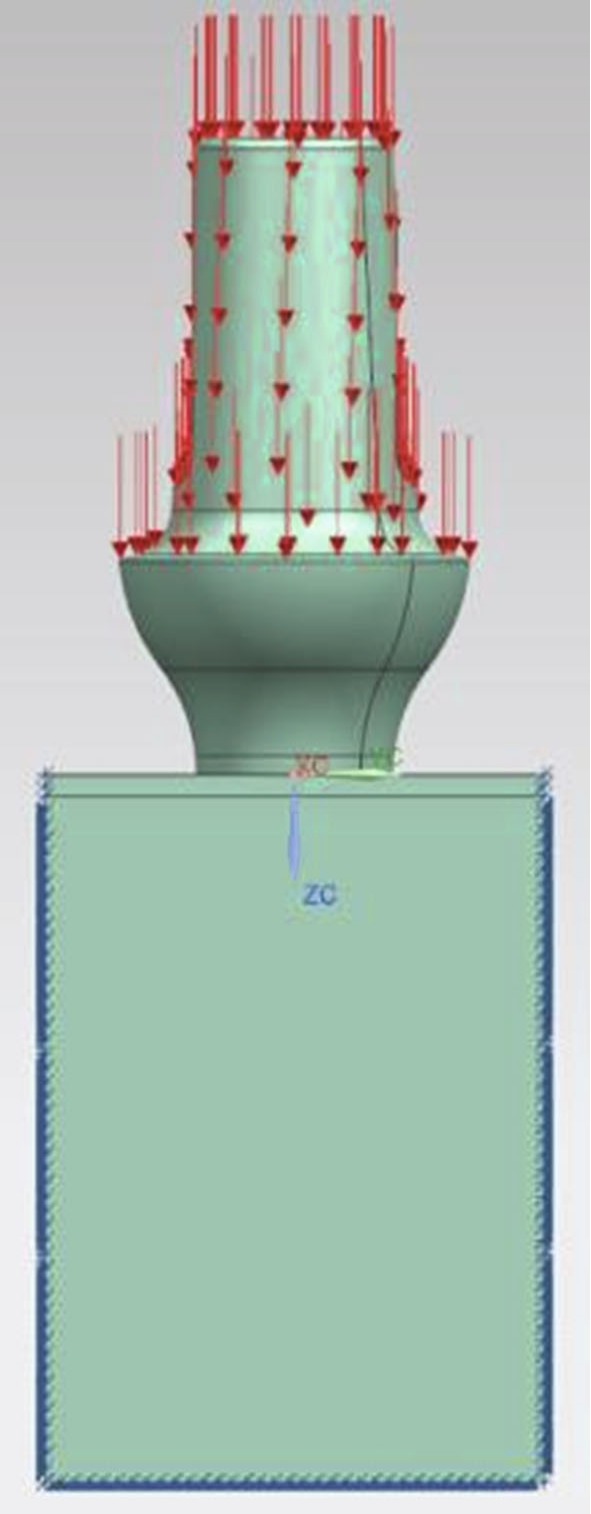
Loading and constraint conditions.

The second force applied to the model is a compression force, equal to 800 N, equally reparted on the apex of the prosthesis. It stimulates the jaw force. The two forces are applied along the *y* axis (Figure 7).

The contacts between the various parts were modeled with nonlinear contact functions. The conditions of contact between bone and prosthesis were considered as “bonded,” to simulate a perfect osseointegration, and therefore a mechanical continuity. As for the contact between the metal surfaces of the prosthesis, they were considered as separate surfaces and in the presence of friction, with a value of the friction coefficient equal to 0.3. The outer surfaces of the bone block have been fixed.

### 2.2. 30-Degree Boundary Conditions

**Figure 8:**
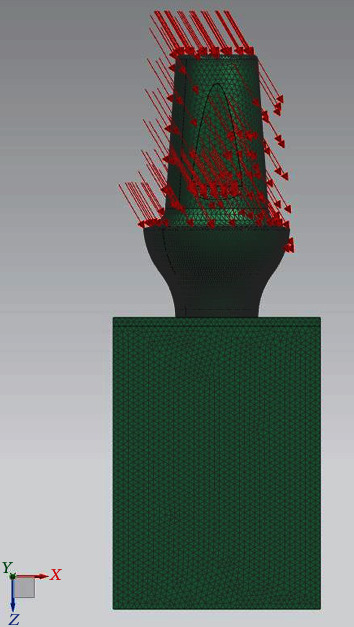
Load direction.

The boundary conditions are the same as in the previous report, i.e., a tightening preload on the screw, the constraints, and the same mesh size. The difference is in the direction of application of the load (800 N); indeed, it has an angle of 30° with respect to the vertical axis *z* (Figure 8).

The material of the dental prosthetic retention and the contacts are the same as in the previous paragraph.

## 3. Results

### 3.1. Zero-Degree Boundary Conditions

The use of finite element analysis allows the evaluations of the stresses that arise in the bone after a prosthetic implant. To perform a correct simulation, the reverse engineering phase is of fundamental importance. The geometric modeling of the prosthesis allows a correct setting of the contacts and frictions and therefore the interactions between the various components of the prosthesis. Another important factor is the inclusion of operating parameters such as the model of the bone tissue material, the preloading of the tightening screw, and the osseointegration of the implant. In Table 3, the maximum equivalent stress value of Von Mises recorded in the prosthesis during the masticatory cycle was inserted. As it is possible to see, the prosthesis reaches a maximum value lower than the titanium yielding stress, thus avoiding plasticization phenomena of the material and above all the static breaking of the prosthesis.

Instead, the values of the stresses acting in the bone tissue are reported. It is possible to see that the maximum stress values are below the static resistance of the bone. In Table 3, instead, the values of the stresses acting in the bone tissue are reported. It is possible to see that the maximum stress values are the static resistance of the bone.

**Figure 9:**
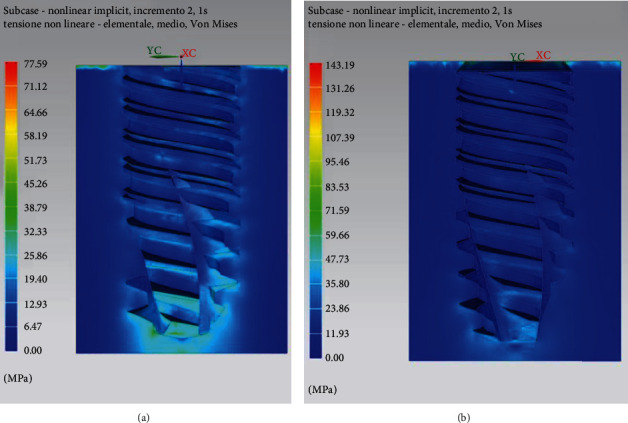
Distribution of stress in cancellous (a) and cortical/cancellous bone tissue. In this second case, two types of bone with different features have been considered (b).

In Figure 9, the trends of the equivalent Von Mises stresses in cancellous and cortical bone tissue are reported. It is possible to see how the distribution of stresses in the bone is located around the implant where the maximum tension is recorded in the cortical bone. With regard to the cancellous bone tissue, the tensions are lower and their distribution on the whole contact surface is generally homogeneous.

**Figure 10:**
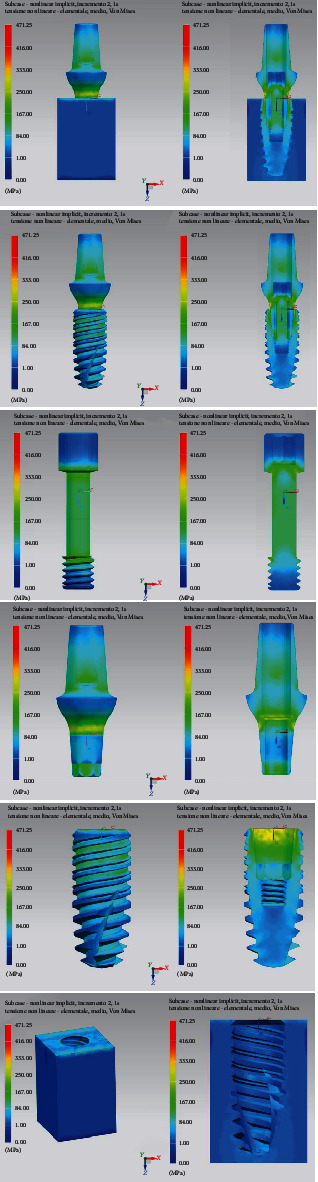
Von Mises tension results in detail.

Figure 10 shows the equivalent Von Mises stresses in detail for each component and its sagittal section. The most stressed components of the entire prosthetic device are the internal tightening screws, with the most stressed areas located at the contact interface between the head of the screw itself and the hole of the abutment; the highest value is recorded in the threaded area in particular in the first fillets. As for the bone implant, here too the distribution is greater in the first three threads.

### 3.2. 30-Degree Boundary Conditions

The use of finite element analysis has allowed to evaluate the stresses that arise in the bone after a prosthetic implant. The extreme operating conditions, due to the force applied at 30°, produce a high concentrated stress on the side of the implant, but always remaining within at about the 90% of the yield condition. This condition is unlikely to happen during the masticatory cycle, but it is convenient to take this into account for precautionary purposes, since its dangerousness. In Table 4, the maximum equivalent stress value of Von Mises was compared with the static resistance value.

In Table 4, the maximum values of the stresses acting on the bone are reported.

**Figure 11:**
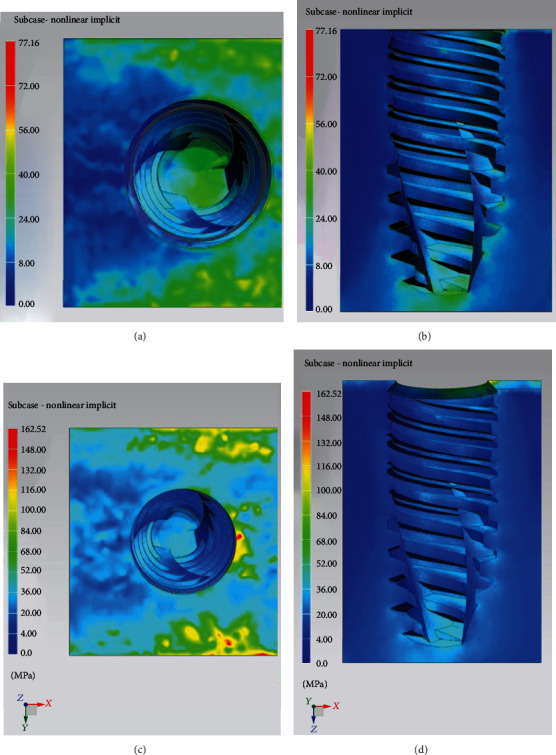
Distribution of stresses in cancellous bone (a, b) and cortical bone (c, d).

In Figures 11(a) and 11(b), the trends of Von Mises stress in the cancellous bone tissue are reported. In particular, it is worth considering the trend of the stresses on the *xy* plane and the sagittal section on the *zx* plane. The stress distribution is influenced by the applied load.

Figures 11(c) and 11(d) shows the stress patterns in cancellous and cortical bone tissue.

**Figure 12:**
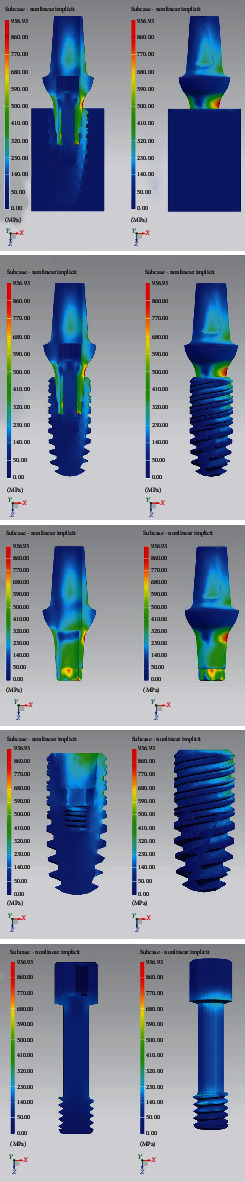
Von Mises tensions at 30-degree results in detail.

Figure 12 shows the Von Mises stresses in detail for each component and its sagittal section. In this case, the most stressed part is the side of the upper abutment, where a lever arm acts that concentrates the stresses.

## 4. Discussion

Finite element analysis (FEM) is a technique that virtually reproduces, from a physical-mechanical point of view, a real condition for studying the interaction between different objects and predicting their mutual behavior under certain load conditions and stress. It is a technique that derives from engineering and is still applied to many fields of the mechanical and aeronautical industry and also in the biomedical sector. Normally, this type of analysis, given its difficulty, is limited to biomedical devices before their industrial production to verify their mechanical properties in relation to their shape. There are various typologies of prosthesis: these are examined by FEM before their production to verify their correspondence with the biomechanical criteria that they must face once implanted [16, 23]. However, the use of FEM has gradually expanded also in the medical sector for the study of mechanically active anatomical parts. Among the applications of finite element analysis in medicine, the simulation of the opening of endovascular stents and the simulation of loads on an orthopedic prosthesis could be made.

The study conducted on this novel dental implant connection therefore includes an analysis of the forces with a load of 800 N at 0 and 30 degrees of angulation, as shown in Figure 12. It is evident however that from the first experiment, the occlusal load provides a release of forces in the apical area of the dental implant, on the medullary bone. In the peri-implant cortex, the distribution of forces appears uniform, without reaching point peaks around the neck of the dental implant. The same applies to the implant components, where the forces are discharged more on the neck of the abutment and on the first turns of the passant screw. In the second experiment, while there are no important differences in the medullary bone, on the cortex this occurs, showing peaks around the neck of the dental implant based on the orientation of the mechanical load, the same is also reflected on the prosthetic components, showing a peak on the hexagon of the abutment and on the neck of the abutment.

Different studies have been conducted using this method on dental implants and implant connections. Based on Kitagawa et al. [24] in comparing different connections, it was found that the external hexagonal joint model had a greater movement than the conical connection model. The external hexagonal model showed a rotation movement, while the movement of the conical connection model showed no rotation. It was concluded that the nonlinear dynamic analysis used in this study clearly demonstrated the rotation differences of the components in dental implant systems with tapering or external hexagonal connections. According to Pournasrollah et al. [25], screw loosening is less likely to occur in the morse hexagonal connection compared to the octagon connection due to the lack of separation of the screw from the internal surface of the abutment.

In the literature, there is a good amount of material that reports FEM studies also carried out in dentistry as an evaluation of the mechanical response of the dental elements subjected to loads of various kinds. In the biomechanical field, the distribution of voltage is analyzed with particular attention, both in biological structures to see how the coupling with an artificial structure (e.g., prosthesis and implant) changes their structural response to external stresses and in artificial structures for check its resistance. The identification in a structure of the distribution and extent of the tensions is important as it highlights which areas are most stressed and therefore most at risk of breaking or, in the case of biological tissues, of necrosis or hypertrophy and which are the areas less stressed which, in the case of biological tissues, could induce atrophy [26–30]. It is necessary to consider that the insertion of implants in the maxillary bones always requires a high precision surgical event, which must be conducted taking into consideration the prosthetic rehabilitation of our patient. Often, the bone or tissue conditions of the jaws do not allow an ideal insertion, and in this case, it may be necessary to conduct further regenerative maneuvers [31, 32]. The innovativeness of this study lies mainly in the fact that all the implant-prosthetic components were evaluated, with a force of 800 N and different angles. The strength of 800 N was taken into consideration following a literature review of previous FEM studies. Furthermore, testing different angles has the purpose of simulating closely the biological conditions created in vivo during chewing. Different studies conducted by Cicciù et al. [1, 15, 22, 33, 34] confirmed the widespread use of finite element methods for calculating the distribution of forces in oral and implant-supported and nonsupported rehabilitations. Knowing the distribution of forces on the implant-prosthetic components is an excellent starting point for improving and modifying the latter in such a way as to respond positively to stresses. From a clinical point of view, it is also useful to know how different angles can affect these components. In fact, implant surgery, which tends to be prosthetically guided, can be further helped by these types of studies.

## 5. Conclusions

The fields of bioengineering come together in the development of new devices for the medical field and medical rehabilitation. Surely, the tested connection shows how studies of this type are able to improve dental implants even before they are fabricated. Being able to perform simulations and thus improve the device is a significant advantage. These new connections guarantee a correct distribution of forces both on the implant components and on the peri-implant tissues. The continuous evolution in the bioengineering field will certainly lead to obtaining ever more performing dental implants.

## Figures and Tables

**Table 1:** Properties of the tested materials.

Properties	Cortical bone	Cancellous bone	Ti6Al4V
Density	1.8 g/cm^3^	1.2 g/cm^3^	4.510 g/cm^3^
Exx	9.6 GPa	0.144 GPa	105 GPa
Eyy	9.6 GPa	0.099 GPa	105 GPa
Ezz	17.8 GPa	0.344 GPa	105 GPa
*ν*xx	0.55	0.23	0.37
*ν*yy	0.30	0.11	0.37
*ν*zz	0.30	0.13	0.37
Gxx	3.10 GPa	0.053 GPa	38.32 GPa
Gyy	3.51 GPa	0.063 GPa	38.32 GPa
Gzz	3.51 GPa	0.045 GPa	38.32 GPa

*E*: elasticity module; *G*: tangential elasticity module, *ν*: Poisson coefficient; each module is reflected in the three space directions.

**Table 2:** Comparison values between the various dimensions of the element.

Element size (mm)	Stress (MPa)	Error (%)
0.1	342.14	/
0.2	326.85	4.47
0.3	299.42	12.49
0.4	274.42	19.79
0.5	253.93	25.78

**Table 3:** The maximum stress Von Mises and Von Mises tension in bone tissue.

	Maximum stress value (MPa)	Static resistance of titanium (MPa)	%
In-Kone® Universal	471.25	1020	53.8%

	Maximum tension on bone tissue (MPa)	Static bone resistance (MPa)	%
In-Kone® Universal	143.19	180	20.45%

**Table 4:** The maximum stress of Von Mises at 30 degrees.

	Maximum equivalent stress (MPa)	Static resistance of titanium alloy (MPa)	%
In-Kone® Universal	936.93	1020	91.8%

	Maximum equivalent tension value on bone tissue (MPa)	Static bone resistance (MPa)	%
In-Kone® Universal	162.52	180	90.3%

## Data Availability

The data used to support the findings of this study are included within the article. The data that support the findings of this study are available from the corresponding author upon reasonable request.
